# Groups of bats improve sonar efficiency through mutual suppression of pulse emissions

**DOI:** 10.3389/fphys.2013.00140

**Published:** 2013-06-13

**Authors:** Jenna Jarvis, William Jackson, Michael Smotherman

**Affiliations:** Department of Biology, College Station, Texas A&M UniversityTexas, TX, USA

**Keywords:** echolocation, sonar, communication, vocalization, noise, bat, acoustic masking, ethernet

## Abstract

How bats adapt their sonar behavior to accommodate the noisiness of a crowded day roost is a mystery. Some bats change their pulse acoustics to enhance the distinction between theirs and another bat's echoes, but additional mechanisms are needed to explain the bat sonar system's exceptional resilience to jamming by conspecifics. Variable pulse repetition rate strategies offer one potential solution to this dynamic problem, but precisely how changes in pulse rate could improve sonar performance in social settings is unclear. Here we show that bats decrease their emission rates as population density increases, following a pattern that reflects a cumulative mutual suppression of each other's pulse emissions. Playback of artificially-generated echolocation pulses similarly slowed emission rates, demonstrating that suppression was mediated by hearing the pulses of other bats. Slower emission rates did not support an antiphonal emission strategy but did reduce the relative proportion of emitted pulses that overlapped with another bat's emissions, reducing the relative rate of mutual interference. The prevalence of acoustic interferences occurring amongst bats was empirically determined to be a linear function of population density and mean emission rates. Consequently as group size increased, small reductions in emission rates spread across the group partially mitigated the increase in interference rate. Drawing on lessons learned from communications networking theory we show how modest decreases in pulse emission rates can significantly increase the net information throughput of the shared acoustic space, thereby improving sonar efficiency for all individuals in a group. We propose that an automated acoustic suppression of pulse emissions triggered by bats hearing each other's emissions dynamically optimizes sonar efficiency for the entire group.

## Introduction

Environmental noise degrades the transmission of all animal communication sounds (Ryan and Brenowitz, 1985; Ryan, 1986; Brumm and Slabbekoom, 2005; Jones, 2008), but echolocation by bats is particularly sensitive because bats need to clearly hear their own faint echoes to hunt and navigate (Neuweiler, 2000; Schnitzler and Kalko, 2001). For bats the most significant source of degrading acoustic interference is the echolocation pulses of other bats, and researchers have long puzzled over how echolocating bats avoid interfering with one other's sonar while flying in dense swarms or within noisy crowded day roosts (Griffin, 1958). In order to echolocate efficiently bats maintain precise control over the acoustic and temporal properties of their echolocation pulses (Neuweiler, 2000; Schnitzler and Kalko, 2001; Schnitzler et al., 2003; Smotherman, 2007), and in some cases this includes adaptations for echolocating in the presence of other bats. Some bats display a jamming avoidance behavior in which they change their outgoing call pitch in order to minimize overlap in bandwidth (Ratcliffe et al., 2004; Ulanovsky et al., 2004; Gillam et al., 2007; Bates et al., 2008; Tressler and Smotherman, 2009; Necknig and Zahn, 2011), and some increase pulse amplitude in the presence of background noise (Simmons et al., 1978; Tressler and Smotherman, 2009; Tressler et al., 2011). These relatively minor changes in pulse acoustics have so far only been documented in pairs of bats and are considered unlikely to be effective for much larger groups of bats because their vocal parameters are tightly constrained by highly specialized laryngeal and respiratory mechanics (Metzner and Schuller, 2007), a finely tuned auditory system (Popper and Fay, 1995), and would force bats to alter pulse characteristics away from optimal parameters for foraging and navigation (Schnitzler and Kalko, 2001). In light of these limitations other more comprehensive answers are needed to explain how bats echolocate in groups.

An alternative to jamming avoidance behavior is for bats to modulate the timing of their pulse emissions to minimize temporal overlap with another bat's echolocation pulses. Many animals acutely regulate the timing of their vocalizations to minimize acoustic interference, including frogs (Loftus-Hills, 1974; Zelick and Narins, 1985; Moore et al., 1989), birds (Ficken and Ficken, 1974; Knapton, 1987; Brumm, 2006; Planque and Slabbekoorn, 2008), and primates (Egnor et al., 2007). Although echolocation serves a different function than these other forms of vocal communication it is possible that bats echolocating in small groups utilize some sort of antiphonal emission strategy to promote emitting pulses out of phase with one another as a means for minimizing temporal overlap with conspecifics, and there is evidence from the field that bats modify emission timing in the presence of other bats (Obrist, 1995). We recently investigated whether solitary free-tailed bats shifted the timing of their pulse emissions in response to artificial acoustic stimuli mimicking the emissions of nearby conspecifics (Jarvis et al., 2010). Bats were found to postpone pulse emissions by roughly 80 ms every time they heard an artificial pulse. We hypothesized that under natural conditions this behavior could promote antiphonal emissions and might also lead to slower pulse emissions in social settings. The potential benefits of antiphonal calling are straightforward, but how this might be managed for even modest sized groups of 5–10 bats is difficult to imagine. Furthermore, if the acoustic suppression of pulse emissions did result in slower pulse emissions for the entire group it was unclear how this could be managed without significantly degrading sonar performance. Here we directly test whether bats emit pulses more slowly in groups than when alone, and if so whether this behavior supports an antiphonal calling strategy that helps bats avoid interfering with one another.

Free-tailed bats are often found hunting insects alone or in small groups of two or three individuals at a particular foraging site, but they also migrate together in dense swarms of tens to thousands of bats and establish day roosts housing hundreds to millions of individuals. In these large densely populated roosts and particularly during emergence from the caves (Gillam et al., 2010) it seems unlikely that any combination of changes in the acoustics or timing could effectively mitigate the interfering effects of the surrounding din. How exactly do free-tailed bats respond to the background noise generated by many continuously echolocating neighboring bats? We predicted that in high population densities free-tailed bats would abandon any attempts to coordinate their temporal emission patterns in favor of emitting pulses more frequently to compensate for information lost due to mutual interference. This was tested using artificial acoustic stimuli simulating the acoustic impacts of progressively larger group sizes.

The results described here indicate that pairs and small groups of 3–10 bats do indeed suppress each other's emissions, but not in support of an antiphonal emission strategy. Instead we find that free-tailed bats appear to adjust pulse emission rates to maximize pulse efficiency, which requires balancing the need to extract more information from the environment by emitting more pulses while minimizing the relative proportion of those pulses producing ambiguous echoes. Drawing upon lessons learned from the study of how information flows through communications networks (Shannon, 1948; Abramson, 1970; Tanenbaum, 2003) we will show how a population density-dependent suppression of pulse emission rates can theoretically improve sonar efficiency in noisy crowded social conditions by improving information throughput of the shared acoustic space. However, when population density grows to the point where the likelihood of an overlap occurring becomes greater than the likelihood of producing an unambiguous echo, the bats switch to emitting pulses at higher rates than when alone. This second strategy may increase the probability of sporadically producing unambiguous echoes or may exploit auditory integration mechanisms that build the auditory scene from bits and pieces of many incomplete or distorted echoes (Moss and Surlykke, 2001; Moss et al., 2006). Free-tailed bats thus adapt their sonar pulse emission rates to differing social contexts via two discreet behavioral responses, slowing pulse emissions to aid coordination in small groups and speeding pulse emissions in dense noisy conditions.

## Materials and methods

### Animals

These experiments utilized captive wild-caught male and female Mexican free-tailed bats (*Tadarida brasiliensis Mexicana)*. All husbandry and experimental procedures were in accordance with National Institutes of Health guidelines for experiments involving vertebrate animals and were approved by the local Institutional Animal Care and Use Committee (TAMU animal use protocol #2007–254). The bats were kept in an artificial habitat with a reversed light cycle and temperature varying daily and seasonally to simulate natural condition. Animals were provided a diet of mealworms supplemented with vitamins and minerals and water was available *ad-libitum*.

### Acoustic recording and playback apparatus

For all experiments bats were placed in a 10 × 10 × 20 cm plastic-coated 1/4″ steel mesh cage which was then positioned in the center of a 6 × 3 × 1.5 meter room lined with sound-absorbing four-inch acoustic foam. The room was kept dark and the temperature was maintained around 30° Celsius during recording sessions. Experiments were performed during the first 4 h after the animals' subjection sunset (12:00–16:00 Zeitgeber time). Vocalizations were recorded with a Brüel & Kjær type 4939 free-field 1/4″ microphone (Brüel & Kjær, Nærum, Denmark) positioned 10 cm from edge of the cage and oriented toward the center. The bats' vocalizations were digitized and analyzed using the hardware and software package Datapac 2K2 (RUN Technologies, Mission Viejo, CA). Pulses were automatically discriminated from background by applying a fixed threshold to the waveform envelope. To account for potential under-sampling due to temporal overlap between simultaneously uttered pulses we visually inspected spectrograms and made corrections by hand as necessary.

Acoustic stimuli were produced with a Vifa 1″ Tweeter (model # BC25SC55-04) powered by a Sony amplifier (model # STR-DE598) which provided a maximum output of ≈80 ± 6 dBs from 15 to 50 kHz. The speaker was mounted 10 cm from and oriented toward the bat's cage. The microphone and loudspeaker were separated by a piece of sound-absorbing foam adjusted daily to minimize the recorded amplitude of the stimulus relative to the amplitude of the bats' pulse emissions. The stimuli for these experiments were digitally created with the TDT OpenEX software v5.4 (Tucker-Davis Technologies, Alachua, FL), and the analog signal was generated by TDT System III RX6 hardware (Tucker-Davis Technologies, Alachua, FL).

### Experiment 1: Do echolocating bats suppress the pulse emissions of their conspecifics?

Individuals or groups of 2–10 naïve bats were recorded echolocating while crawling around the steel mesh cage positioned in the center of the anechoic recording chamber. The mean pulse emission rate per bat was calculated as the total number of pulses detected divided by total duration of the recording and the number of individuals placed in the cage. To determine whether an artificial stimulus altered pulse emission rates solitary bats were presented with artificial downward frequency-modulated sounds mimicking the echolocation pulses of free-tailed bats (Jarvis et al., 2010) at a repetition rate of five pulses per second, similar to naturally behaving bats.

### Experiment 2: Does mutual suppression lead to reduced incidences of overlapping pulse emissions?

To determine whether the prevalence of overlapping pulse emissions occurred less frequently than predicted based on random chance we compared the real rate of overlaps occurring between two bats with Monte Carlo simulations of pairs of bats echolocating together. Real rate of overlaps was measured by manually counting the numbers of overlapping pulses occurring in randomly selected 10-s time epochs collected from 141 separate recordings of pairs of bats. We defined an overlap event as any instance when a second pulse appeared in the spectrogram within 10 ms of the onset of a previous pulse. Pulse durations typically varied from 4 to 8 ms and the returning echoes perpetuated in the chamber for at least 5 ms beyond the end of the first pulse. Under natural conditions the period over which another bat's emissions might overlap with the time course of a returning echo likely extends well beyond the 10 ms limit used here, but we will show that the results presented here are easily adapted to reflect more liberal time windows to accommodate different species or habitats. Monte Carlo simulations of pairs of bats echolocating together were generated using 100 randomly chosen 10-s epochs of acoustic recordings from isolated naïve bats, which gave 4950 discreet simulated cross-pairings. For each real and simulated epoch we measured the mean pulse rate and number of overlaps occurring within the 10 s epoch and from this determined the probability distribution of overlaps as a function of mean pulse rate. It was not possible to discriminate between the echolocation pulses of real bats recorded in pairs reliably enough to measure each individual bat's pulse emission rate. Finally, based on the assumption that simultaneous emissions always have the potential to create ambiguities in the perception and interpretations of succeeding echoes, we define *pulse efficiency* as the mean proportion of emitted pulses that did not overlap with another bat's emissions and therefore likely produced unambiguous echoes. Pulse efficiency was calculated by subtracting the expected interference rate (overlaps per second) from mean pulse emission rate.

### Experiment 3: How do bats respond to the presence of continuous noise?

To measure the behavioral response to continuous noise we measured the effects of a prolonged broadband noise stimulus on pulse emission rates. Preliminary experiments indicated that the bat's pulse emission rates typically declined over the 20–30 min time-course of an experimental session regardless of stimulus type, preventing us from directly comparing extended recordings of bats echolocating in noisy vs. silent conditions. Furthermore, individual call rates varied significantly across days, making it difficult to achieve statistically significant results when comparing stimulus conditions across days. Therefore, to control for daily fluctuations and the systematic short-term decline in emission rates seen over the course of initial recordings, bats were exposed to a time-varying noise stimulus composed of 10-s blocks of white noise alternated with 10-s of silence. An iterative process led us to compromise upon 10-s stimulus epochs because this timeframe was at least two orders of magnitude longer than their typical inter-pulse intervals and yet short enough that there was no detectable time-dependent reduction in mean call rate within each epoch. Preliminary trials with longer epochs of up to 2 min produced qualitatively similar results. This stimulus pattern will hereafter be referred to as the “continuous” noise stimulus to distinguish it from the periodic noise-burst stimuli used in Experiment 1 and our previous study (Jarvis et al., 2010). For each trial the total number of echolocation pulses uttered was pooled from all experimental (stimulus ON) and silent (stimulus OFF) conditions and both mean emission rate and relative proportion of pulse's uttered was calculated for the noise On and noise Off conditions. To test if the bats responded differently to noise when alone vs. in the presence of other bats, experiments were conducted in two separate sessions. In the first session, recordings were carried out with groups of either four or eight bats placed in the same cage and collectively exposed to the continuous noise stimulus. Following this, each bat from the group was isolated and recorded individually while being exposed to the same series of stimuli. Data were normalized as the total percentages of pulses occurring in silence vs. noise.

### Experiment 4: At what temporal ratio of noise to silence does the noise promote faster emissions?

Six solitary bats were exposed to stimuli of varying duty cycles constructed by alternating a 10 ms burst of broadband noise with silent intervals of variable length. For example 10 ms of noise alternating with a 90 ms silent period gave a 10% duty cycle; other silent intervals were 40 ms (20% duty cycle), 10 ms (50% duty cycle), 3.3 ms (a 75% duty cycle), and 1.1 ms (a 90% duty cycle). Each bat was recorded for six 12-min exposures to each duty cycle. During these recording sessions, the stimulus was switched on and off every 2 min, allowing the stimulus blocks to be interspersed with blocks of silence. The total number of echolocation pulses uttered was pooled from all 6 min of experimental (stimulus ON) and silent (stimulus OFF) conditions during each session. Different duty-cycle stimuli were presented in pseudorandom order to balance for time and order effects.

### Statistical analysis

All result are expressed as mean ± standard deviation. Statistical analyses were performed with Sigma Stat v.9.0 (Systat Software, San Jose, CA). For Experiment 1 non-parametric *t*-tests and a Kruskal-Wallis One-Way analysis of variance on ranks was used to investigate the effect of population density on average pulse rate, and a least-squares method was used to determine the best curve fit. For Experiments 2 and 3, a Two-Way analysis of variance test was performed to investigate the effects of noise and social conditions on pulse emission rates. For Experiment 4, a Two-Way analysis of variance using Holm-Sidak multiple comparison tests was performed to determine the effects of stimulus condition and duty cycle on emission rates.

## Results

### Experiment 1: Do echolocating bats suppress the pulse emissions of their conspecifics

**Figure 1:**
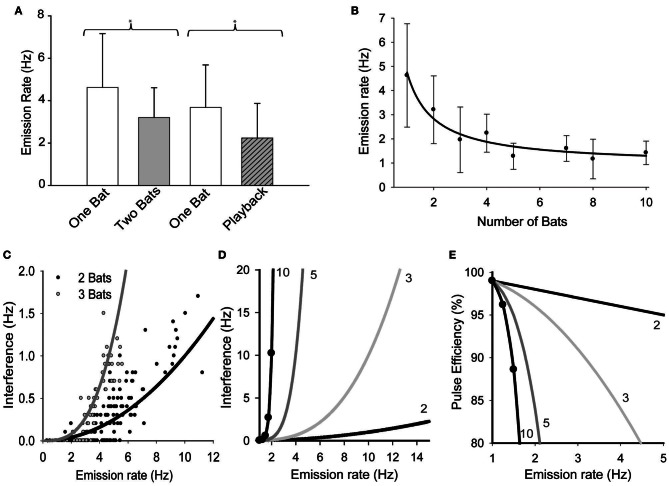
**The effect of group size on pulse emission rates. (A)** Bats' mean pulse emission rates recorded alone vs. when echolocating in pairs, and then again for alone vs. while echolocating with a speaker simulating the presence of another bat echolocating (playback). **(B)** Average emission rates per bat plotted vs. the total number of bats in the group. Pairwise multiple comparisons indicated that mean pulse emission rates for groups of 3 or more bats were significantly lower than solitary bat emission rates (*Q* = 5.033, *p* < 0.05). Data were fit with a first order linear regression (solidline, *y* = 0.92 + 3.82/*x*). **(C)** Plot of mean pulse rates vs. the rate at which overlaps occurred (interferences) for pairs (*n* = 141) and triads (*n* = 56) of bats. Both sets of data were well fit by the same simple power function of the form *y* = *r*τ^*n*^, where *r* = mean emission rate (Hz), τ = overlap window duration (ms) and *n* = number of bats. [*r*^2^= 0.71, *F*_(1, 140)_ = 344.9, *P* < 0.001]. Extending the functions derived from **(C,D)** illustrates the expected effect of pulse emission rates on mutual interference rates for groups of 2, 3, 5, and 10 bats. **(E)** These functions were then used to predict the effect of pulse emission rates on the proportion of pulses expected to generate unambiguous echoes, or *y* = 1 − *r*τ^*n*^ (pulse efficiency) for different group sizes.

There was a significant reduction in mean emission rates when bats were echolocating in pairs vs. when they were alone (Figure 1A, Mann-Whitney test. *T* = 930, *n*_1_ = 28, *n*_2_ = 57, *p* = 0.011). There was also a significant reduction in pulse emission rates when bats echolocated while the loudspeaker played back an artificial stimulus mimicking the presence another free-tailed bat (Figure 1A; *t* = 2.045, *df* = 35, *p* = 0.048). Figure 1B plots the significant effects of increasing bat density on the mean pulse emission rates (*H* = 90.199, *df* = 7, *P* = 0.001). The negative relationship between bat density and mean pulse emission rate was best fit by an inverse first order non-linear regression [*F*_(1, 6)_ = 93.97, *p* < 0.0001, *R*^2^ = 0.94] that decayed toward an asymptote equivalent to ~20% of the mean emission rates for naïve solitary bats, or roughly 1 pulse per second.

### Experiment 2: Does mutual suppression lead to reduced incidences of overlapping pulse emissions?

Comparing real groups of bats to Monte Carlo simulated groups of bats revealed that the bats' echolocation behavior was strongly altered by social context. Real pairs of bats emitted significantly fewer pulses per second than simulated pairs (4.6 ± 2.1 Hz, *n*=141 vs. 6.0 ± 3.1 Hz, *n* = 4950, respectively, *P* < 0.0001) and also emitted overlapping pulses significantly less frequently than simulated pairs (0.29 ± 0.37 Hz vs. 0.38 ± 0.38 Hz, *P* < 0.0001). Analyses also revealed that real pairs produced a higher percentage of epochs with no instances of overlap (48%) than simulated pairs (15%) suggesting that real pairs of bats were successfully avoiding overlaps better than expected by chance alone. However, this observation could simply be a product of reduced pulse emission rates, since the number of overlaps per second was strongly correlated with mean pulse emission rates per epoch for both real and simulated bats (*R* = 0.83, *p* < 0.0001 and *R* = 0.75, *p* < 0.0001, respectively). To investigate this we examined whether the reduction in interferences was independent of pulse emission rates. It was hypothesized that if bats actively avoided overlapping with one another's emissions, then the data from real bats should reflect a change in the correlation between interference rates and pulse emission rates. This was found not to be true; although real pairs of bats emitted fewer pulses per second neither the mean overlap rate nor the slope of the correlation varied significantly over the overlapping range of emission rates (*P* > 0.05). Alternatively if the probability of two or more bats' emissions overlapping in time was random, then the interference rate was predicted to follow a simple power function of the form *r*τ^*n*^, where r is the mean emission rate, τ is the empirically defined overlap window duration (10 ms), and *n* is the number of bats. Figure 1C plots how frequently real bats echolocating in pairs or triads emitted overlapping pulses (labeled *Interferences*, quantified as overlaps per second) as a function of the mean pulse emission rate. Both data sets were well fit by the function *r*τ^*n*^ [*r*^2^= 0.71, *F*_(1, 140)_= 344.9, *P* < 0.001], indicating that interferences had occurred randomly and their propensity was predictably based on mean emission rates and population density and that the bats were not timing their pulse emissions to avoid overlaps with one another. Figure 1D extends this function to illustrate how pulse emission rates are predicted to influence interference rates for groups as large as 10 bats. The graph demonstrates that bats in modest group sizes of five or more are faced with a daunting increase in the probability that their pulse emission will overlap with those of neighboring bats. Figure 1E uses the same functions to estimate pulse efficiency (1 − *r*τ^*n*^) as a function of pulse emission rate. This provides an estimate of the relative proportion of emitted pulses that would likely return unambiguous echoes over a natural range of pulse emission rates, illustrating that pulse efficiency is expected to decrease steeply with increasing population density and faster emission rates.

### Experiment 3: How do bats respond to the presence of continuous noise?

**Figure 2:**
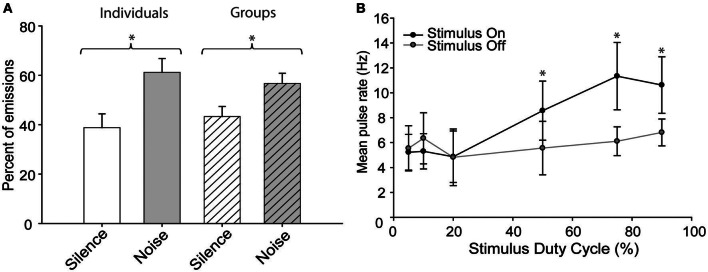
**The effect of continuous noise stimuli on pulse emission rates. (A)** Bats emitted pulses more frequently in the presence of continuous background noise than during intervening silent periods. The effect was similar whether recording from individuals or groups of bats. **(B)** The effect of stimulus duty cycle on the mean pulse emission rates of solitary bats. Error bars indicate standard deviation; asterisks indicate statistically significant differences from intervening silent periods (*P* < 0.01).

When exposed to “continuous” blocks of broadband noise, the bats emitted pulses more frequently while the noise was present than during the intervening silent periods (Figure 2A) regardless of whether they were recorded individually or in groups [Two-Way ANOVA, *F*_(1, 40)_ = 143.8, *p* = 0.001]. There was also a significant interaction effect between the social and noise conditions [*F*_(1, 40)_ = 8.937, *p* = 0.005] arising because bats called more frequently in noise than silence but less frequently in groups than alone, indicating that these effects were combinatorial and not mutually exclusive. Social condition had no significant effect upon the response to sustained noise stimuli. The mean pulse emission rates were lower for groups vs. solitary conditions but increased in noise under both conditions (group rates were 1.5 ± 0.9 Hz in silence vs. 1.8 ± 1.3 Hz in noise; solitary rates 1.8 ± 0.8 Hz vs. 2.3 ± 1.0 Hz in noise). Although the general behavior was consistent with previous results the overall range of pulse emission rates during these experiments was less than in earlier experiments because the bats were no longer naïve to the recording chamber and had habituated to the experimental procedure.

### Experiment 4: At what temporal ratio of noise to silence does the noise promote faster emissions?

The above experiments demonstrate that free-tailed bats respond differently, depending on whether the interfering noise stimulus is continuous or periodic. Specifically, bats emit pulses less frequently in periodically noisy conditions but more frequently in the presence of sustained noise. To better estimate the point at which bats treat a noise as continuous vs. periodic, a subset of bats were exposed to a series of noise burst stimuli presented at duty cycles ranging from 5 to 95% and we compared pulse rates during stimulus presentations to the rates obtained during intervening silent periods (Figure 2B). Stimulus duty cycle had a significant effect upon pulse emission rates [Two-Way ANOVA, *F*_(1, 70)_ = 14.888, *p* = 0.001] with was a statistically significant interaction effect between the noise status (on/off) and stimulus duty cycle [*F*_(5, 70)_ = 5.123, *p* = 0.001]. *Post-hoc* tests determined that while there was no significant difference in pulse rates among the 5, 10, and 20% duty cycle conditions, duty cycles at or above 50% caused a significant increase in pulse emission rates relative to silent conditions [Holm-Sidak method; 50%, *t* = 2.652, *p* = 0.05; 75%, *t* = 4.613, *p* = 0.05; 90%, *t* = 3.355, *p* = 0.05; *F*_(5, 70)_ = 8.872, *p* = 0.001]. There was no significant difference in emission rates across duty cycles at or above 50%, indicating that the bats responded similarly to all of these stimuli as if they were continuous noise.

## Discussion

Mexican free-tailed bats live in large dense colonies consisting of hundreds to millions of individuals (Simmons et al., 1978; Ratcliffe et al., 2004). They are highly social animals that spend a large part of their time echolocating in close proximity to other echolocating bats. It is assumed that high population densities present significant challenges for an active sonar system, since signal degradation and perceptual ambiguities are expected to arise from interferences derived from other bats' echolocation pulses. Whether or not bats utilize behavioral strategies for mitigating this interference is unknown. We previously reported that free-tailed bats responded to brief noise bursts by postponing the emission of subsequent echolocation pulses (Jarvis et al., 2010). We speculated that this behavior might improve sonar performance in social conditions by encouraging an antiphonal emission strategy among pairs or small groups of bats. The results presented here dismiss that hypothesis, instead demonstrating that the suppression caused by hearing one another's pulses does not lead to temporal coordination of pulse emissions among pairs or triads of bats. Monte Carlo simulations support the conclusion that overlaps occurred randomly and pairs or triads of bats performed no better than chance at avoiding overlap with each other's emissions.

It was also hypothesized that the acoustic suppression of pulse emission might lead to the generalized suppression of pulse emissions in groups. This was confirmed. Bats slowed their pulse emission rates in response to hearing either the echolocation pulses of real bats or artificial echolocation pulses. Increasing bat density resulted in greater suppression of emissions, indicating that the suppressive effects were additive in nature. If neighboring bats suppress each other's pulse emissions but this suppression does not promote an antiphonal emission strategy, what then is the benefit of this behavior? Here we propose that lessons learned from modern communications networks may explain how slowing pulse emissions can improve a bat's sonar performance when echolocating within a group.

The *ALOHA* system was an inaugural experiment in computer networking designed to link multiple independent users spread across the Hawaiian Islands to a central mainframe computer via a shared UHF radio channel (Abramson, 1970). Signals were randomly transmitted to and from a central computer in time-limited bursts or “packets” of information in a completely unsynchronized manner which led to “collisions” among users transmitting at the same time, causing the loss of both signals. Error detection algorithms were instituted that allowed users to know when their signals had collided, and a simple re-transmission protocol was incorporated independently by users that continually resent signals until a successful transmission occurred. This resulted in an uncoordinated competition for channel time that degraded the overall flow of information for all users. To improve network efficiency ALOHAnet's architects investigated how often collisions occurred and how to best to guide user behavior to optimize information flow through the network while also improving transmission efficiency for each user (Abramson, 1970). Network performance was characterized by its total information *throughput* as a function of overall *traffic load*.

**Figure 3:**
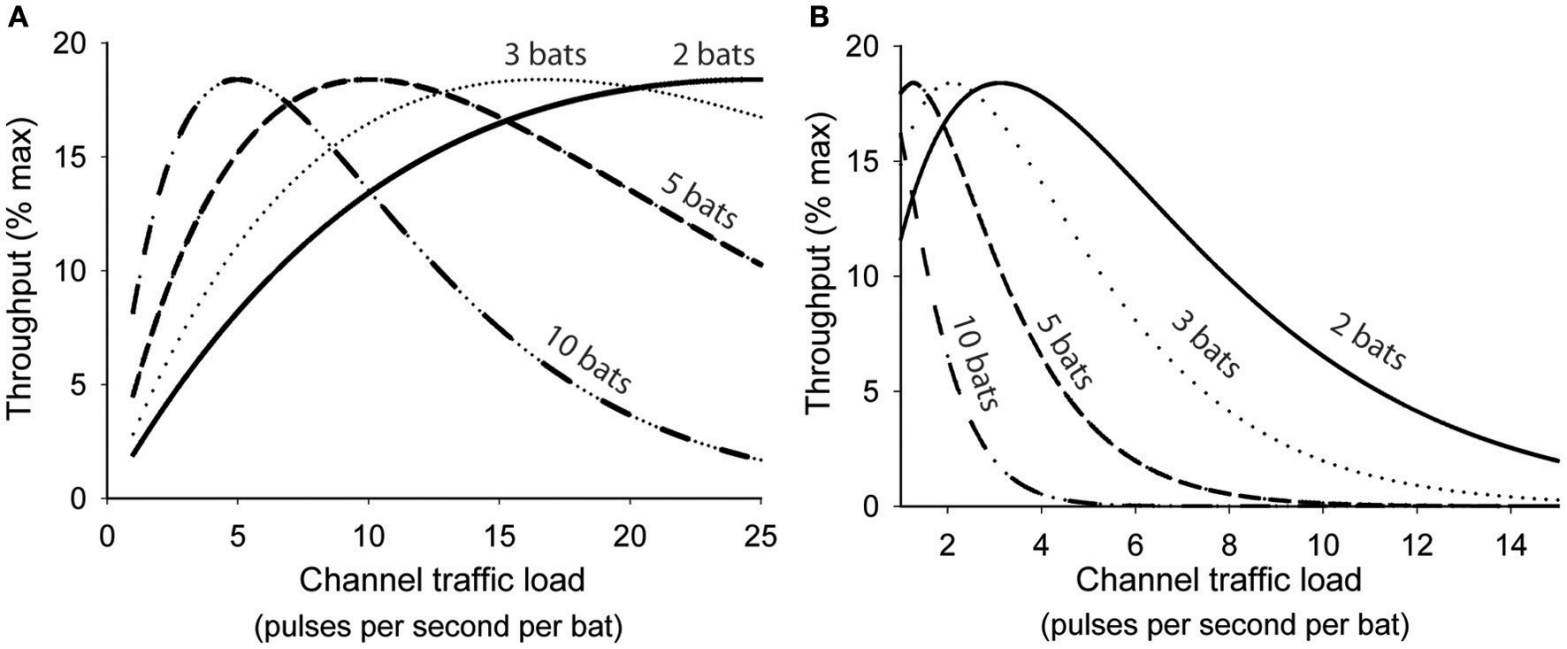
**Interactive effects of population density and emission rates on theoretical information throughput (*S*) of a shared acoustic communication channel following the function *S* = λ*e*^2λ^, where λ = *r*τ^*n*^. (A)** calculates information throughput assuming a conservative overlap window (τ) of 10 ms while **(B)** assumes an empirically-determined overlap window of 80 ms. In both graphs throughput is maximized at progressively slower emission rates as group size increases. In B the peak S is achieved at an optimum emission rate 3.25 Hz/bat for pairs of bats, 2.0 Hz/bat for triads, 1.25 Hz/bat for groups of five, and 1 Hz/bat for groups of ten.

Abramson and colleagues showed that as channel traffic increased the rate of collisions among user transmissions increased exponentially and consequently the probability of a successful transmission decreased exponentially (Abramson, 1970). For any single user the immediate probability (*p*) of a successful transmission was predicted by *p* = *e*^−2λ^, where λ was a product of the number of users (*n*), mean transmission rate (*r*), and signal duration (τ). Channel throughput (*S*) was used as a measure of how efficiently information is transmitted through a shared communication channel. Maximum possible throughput for any shared channel is achieved only when all user transmissions are perfectly coordinated to utilize 100% of the channel time without any collisions, and is effectively unachievable without comprehensive central coordination. Since a channel's capacity to transmit information can also be underutilized, *S* is ultimately a function of both channel usage and *p*, thus *S* = λ*e*^−2λ^, reflecting the compromise between transmission rate and interference rate. Figure 3A illustrates how this function could be applied to a group of bats sharing a common acoustic space, except that in this analogy the acoustic space represents a shared communication channel. All the bats sharing the space are transmitting and receiving their echolocation pulses over the same shared channel, and each bat is likely to lose information when its transmissions collide with another bat's transmissions. For analytical purposes we assume that any overlapping pulse emissions result in the total loss of both transmitted signals, but this may not be entirely true for bats. For free-tailed bats we define *r* = mean pulse emission rate, τ = overlap window (10 ms), and then λ = *n*_bats_ *r*τ. For any given population density greater than one it can be shown that there is an optimum mean pulse emission rate where all bats would presumably benefit from increased pulse efficiency, deriving the most information possible from their echolocation pulse stream with the least amount of wasted emissions. Increasing pulse emission rates beyond this optimum rate rapidly degrades information throughput of the common airspace because the relative proportion of pulses generating unambiguous echoes steeply declines for all individuals.

The random-access nature of a “pure ALOHA” network such as the one described above was found to constrain network throughput to a maximum value of 0.5/e, or roughly 18.4% of the theoretical maximum achievable capacity (Abramson, 1970; Kleinrock and Tobagi, 1975). Since interferences automatically trigger re-transmissions, such random-access networks are inherently unstable due to a positive feedback loop wherein retransmissions lead to a progressively increasing traffic load and consequently more frequent collisions or interferences. For bats, this means that if all the animals in the group increased pulse emission rates to compensate for lost information due to mutual interference, as might be expected based on their known response to cluttered acoustic environments (Petrites et al., 2009), then their net sonar performance would decline rather than improve. Instead, to maintain even modest throughput efficiency bats would be better off reducing emission rates as *n* increased, else the number of pulses generating unambiguous echoes would rapidly diminish. To combat this phenomenon in ALOHAnet, regulatory protocols were applied to constrain when and how often users retransmitted their data. One of these, known as the “carrier sense multiple access” protocol (CSMA) is relevant to bats because CSMA incorporated a “listen-before-send” algorithm, in which transmitters first checked to see if the channel is free before transmitting, and if not briefly postpone transmissions. This greatly reduced traffic load by reducing the number of collisions and retransmissions, and thereby increased network utilization and information flow for all users. We now hypothesize that acoustic suppression of pulse emission exhibited by free-tailed bats serves a function similar to CSMA in wireless communication networks, effectively improving sonar performance in social settings by optimizing pulse emission rates relative to population density.

The optimum range of pulse emission rates predicted by Figure 3A is significantly higher than the emission rates we observed for similarly sized groups of bats (Figure 1B). This may be accounted for by differences in the predicted and actual overlap window durations. We used a conservative estimate of 10 ms in our analyses, however, our previous studies indicate that hearing another bat's echolocation pulses can suppress echolocation pulses for up to 80 ms, suggesting that the effective overlap window is somewhere closer to 80 ms. The actual time window over which returning echoes may be subject to interference should vary predictably with habitat and target distances, but it is possible that in free-tailed bats the general behavior is tuned to a specific range, represented by an echo delay of 80 ms. When we recalculated information throughput values using an 80 ms value for τ (Figure 3B) we found optimum pulse emission rates more closely aligned with the empirically obtained emission rates for groups of different sizes. This supports the hypothesis that free-tailed bats are reducing their pulse emissions to optimize information throughput of their shared acoustic channel.

Importantly, pulse emissions were never entirely suppressed. At group sizes of five or more the emission rates approached an asymptotic minimum of ~1 Hz, equivalent to about 20% of the average pulse rate of solitary bats under identical conditions. This indicates that pulse emissions would never be entirely suppressed by the echolocation pulses of their neighbors regardless of population density. In fact, in contrast to the suppression caused by brief periodic noise bursts, we found that sustained broadband noise increased pulse emission rates. This effect was evident regardless of whether bats were alone or echolocating in groups. Pulse emission rates only increased significantly at stimulus duty cycles greater than or equal to 50%, leading us to conclude that once the noise occupies more than half the available time window they behaved as though the noise was essentially continuous. This is consistent with the idea that once the probability that an emitted pulse will overlap with noise exceeds 50%, the bats behave as though every echo may be compromised by noise. Emitting more pulses per second when echolocating in a constantly noisy environment might increase the probability of sporadically producing unambiguous echoes and may improve echo perception via cognitive mechanisms that allow for integration of auditory cues over many sequential echoes, thereby building a more accurate perceptual map of the auditory scene from bits and pieces of many incomplete or distorted echoes (Moss and Surlykke, 2001; Moss et al., 2006).

## Conclusion

Solitary bats normally resolve ambiguities in their auditory scene analyses by speeding up their pulse emission rates (Moss et al., 2006; Petrites et al., 2009). Here we propose the counterintuitive hypothesis that echolocating bats cooperatively optimize sonar performance at the group level by *slowing* their pulse emission rates proportional to population density, mirroring protocols developed to optimize information throughput in artificial communications networks (Abramson, 1970). Conspecific bats sharing the same acoustic space must transmit and receive their sonar emissions over a single shared communication channel and therefore face many of the same challenges that constrain wireless communications networks. In artificial systems channel capacity is optimized by regulating the transmission behaviors of users via a common set of rules and constraints that ultimately improves efficiency for all users (Tanenbaum, 2003). Likewise, echolocating bats may have evolved a transmission-delay algorithm similar to those used in communications networks to optimize sonar performance in social contexts. Since these experiments were done with stationary bats, it remains to be seen whether flying free-tailed bats performing challenging sonar-guided navigational tasks also display this behavior, though there is evidence from the field and the lab showing that other species of bats increase inter-pulse intervals in the presence of other bats (Obrist, 1995; Chiu et al., 2008). During flight pulse emissions are significantly constrained by additional mechanical and physiological factors not present when stationary. From a theoretical standpoint, however, flying bats should have as much if not more to gain as stationary bats from exploiting this strategy. The principle that sometimes less is more may prove to be an important clue toward understanding how bats echolocate together in large groups.

### Conflict of interest statement

The authors declare that the research was conducted in the absence of any commercial or financial relationships that could be construed as a potential conflict of interest.
